# Biomacromolecules, Biobased and Biodegradable Polymers (2017–2019)

**DOI:** 10.3390/polym12102386

**Published:** 2020-10-16

**Authors:** Naozumi Teramoto

**Affiliations:** Department of Applied Chemistry, Faculty of Engineering, Chiba Institute of Technology, 2-17-1 Tsudanuma, Narashino, Chiba 275-0016, Japan; teramoto.naozumi@it-chiba.ac.jp; Tel.: +81-47-478-0406

**Keywords:** biomacromolecule, biopolymer, biobased polymer, biodegradable polymer, environmentally benign material, biomaterial

## Abstract

Now, we have over 1000 papers in the field of “Biomacromolecules, Biobased and Biodegradable Polymers”, one section of *Polymers* (*Basel*). This is one of the largest sections in *Polymers*, including issues on biomacromolecules, biobased polymers, and biodegradable polymers for applications with environmentally benign materials, biomedical materials and so on. These applications are attracting attention day by day as there exist a lot of problems regarding environmental and biomedical issues. Here I reviewed papers published in this section between 2017 and 2019 and introduce prominent papers, analyzing the numbers of citations (times cited).

## 1. Introduction

*Biomacromolecules* and *biopolymers* are narrowly defined as polymers derived directly from living organisms such as carbohydrate polymers, proteins, nucleic acids and lignin. Since researchers have produced various types of polymers using biomass as chemical resources for several decades, this movement has widened the interpretation of the terms *biomacromolecules* and *biopolymers*. For example, poly(lactic acid) (PLA) is synthesized chemically by the artificial polymerization of lactic acid, which is a fermentation product of lactic bacteria. According to the narrow definition, PLA is not classified as a biomacromolecule or biopolymer. On the other hand, poly(3-hydroxybutyrate) (PHB) can be produced directly by the fermentation of bacteria such as *Ralstonia eutropha* (formerly known as *Alcaligenes eutrophus*), *Azohydromonas lata* (formerly known as *Alcaligenes latus*), *Pseudomonas oleovorans* and recombinant *Escherichia coli* on medium containing glucose or glycerol. Both PLA and PHB are thermoplastic aliphatic polyesters, and the repeating unit differs only by one methylene group, as PLA can be described as poly(2-hydroxypropionate). Both PLA and PHB are known to be applied to medical use because of their biodegradable behavior in mammalian bodies. In such a context, PLA has become classified as a biomacromolecule and biopolymer. As another example, chemically synthesized modified biopolymers such as artificial polypeptides synthesized by the polymerization of unnatural amino acids have been classified as biomacromolecules and biopolymers, because they can be assumed to be modified polypeptides judging from their chemical structures. Today, the expanded interpretation of biomacromolecules and biopolymers covers most biobased polymers having similar structures of naturally derived polymers.

*Biobased polymers* are classified as polymers whose raw materials are derived from biomass, or polymers that are produced by living organisms (narrowly defined *biopolymers*). The word intimates the meaning of polymers from renewable sources under environmental consciousness and sustainability. The term *biodegradable polymers* literally means polymers that can be degraded by biochemical processes or simple hydrolysis. More practically, this category contains two kinds of polymers: (1) polymers that can be degraded by microorganisms distributed widely in nature and (2) polymers that can be degraded in mammalian bodies. The former polymers are related to environmentally benign materials, and the latter, related to biomaterials.

At present (September 2020), our journal *Polymers* (*Basel*) has 10 special sections: Polymer Chemistry; Polymer Analysis; Polymer Physics; Polymer Theory and Simulation; Polymer Processing and Performance; Polymer Applications; Biomacromolecules, Biobased and Biodegradable Polymers; Polymer Recycling; Polymer Composites and Nanocomposites; and Green and Sustainable Chemistry in Polymer Science, excepting the General section. Our section “Biomacromolecules, Biobased and Biodegradable Polymers” has over 1000 articles, placing it second among these sections in rank by the number of articles. I searched papers published in this section between 2017 and 2019, and here introduce prominent papers related to this section. In the section “Biomacromolecules, Biobased and Biodegradable Polymers”, we can divide all the papers into three categories: (1) addressing polymers for biomaterials and biomedicine, (2) addressing environmentally benign polymers, and (3) addressing biomacromolecules for other applications or the general science and technology of biomacromolecules (whose applications are not limited). We can find a lot of papers categorized into (1) and (2), while the number of papers categorized into (3) is not so large.

## 2. Category (1): Polymers for Biomaterials and Biomedicine

In this category, we often see two important keywords, “drug delivery” and “wound dressing”, which reflect the current topics of polymers for biomaterial applications. Among related polymer resources, “chitosan” is predominantly picked up for these applications. The title of the most cited paper that we checked in September 2020 is “Chitosan and Its Derivatives for Application in Mucoadhesive Drug Delivery Systems”, which is a review paper co-authored by Twana Mohammed M. Ways, Wing Man Lau and Vitaliy V. Khutoryanskiy [1]. The number of citations as analyzed by Web of Science is 87 (on 22 September 2020). They carefully collected and introduced research related to mucoadhesive drug delivery applications of chitosan and chemically modified chitosan, referring to 165 papers. Chitosan is a hydrophilic polysaccharide derived from chitin by the hydrolysis of acetamido groups. Its main repeating unit is (anhydro-)d-glucosamine, while (anhydro-)*N*-acetyl-d-glucosamine units partially remain in the chain. Chitosan can be charged positively, and this charge can play an important role in the binding of chitosan and mucus.

According to rank by citation number, “Chitosan Combined with ZnO, TiO_2_ and Ag Nanoparticles for Antimicrobial Wound Healing Applications: A Mini Review of the Research Trends”, which is a review paper co-authored by Vu Khac Hoang Bui, Duckshin Park and Young-Chul Lee [2], comes next (the citation number: 61). The antimicrobial ability of wound dressing materials is important for preventing infection by harmful bacteria. Though chitosan is known to have antimicrobial ability, it is not enough because of dependency on the pH and classes of bacteria. Therefore, the combination of chitosan and other antimicrobial substances is needed to enhance the antimicrobial ability. The authors focused on ZnO, TiO_2_ and Ag nanoparticles as filler substances for chitosan-based wound healing materials in this review.

As the most cited research article (other than review papers), we should select the paper entitled “Electrospun Polycaprolactone/Aloe Vera_Chitosan Nanofibrous Asymmetric Membranes Aimed for Wound Healing Applications” co-authored by Sónia P. Miguel, Maximiano P. Ribeiro, Paula Coutinho and Ilídio J. Correia [3] (the citation number: 48). The advantage of the material reported in this paper is an asymmetric membrane with a two-layer structure. They devised a two-layer structure composed of a top layer of electrospun polycaprolactone (PCL) and a bottom layer of an electrospun membrane prepared from chitosan and aloe vera gel. These two latter components, chitosan and aloe vera gel, are both known to be efficient for wound healing. The PCL top layer can play a role as a protective layer for human skin (keratinous layer).

Indeed, we can see the word “chitosan” in the titles of papers that attract citations. Javier Pérez Quiñones et al. [4] reviewed technical methods for the preparation of chitosan nanoparticles by self-assembly (the citation number: 49). Through a lot of tables, they depicted the relationship between the procedure/active agent and particle size/zeta-potential. Dongying Zhao et al. [5] summarized chitosan and its derivatives for biomedical applications, especially focusing on nanoparticle applications (the citation number: 46). Gheorghe Adrian Martău et al. [6] summarized chitosan, alginate and pectin from a wider point of view of food, biomedical and pharmaceutical applications (the citation number: 21). They especially focused on the biocompatibility, biodegradability and bioadhesiveness of these biopolymers. Chitosan is a polycation in the presence of a counter anion, and it can bind to polyanions such as DNA and RNA under acidic conditions. Beatriz Santos-Carballal et al. [7] overviewed the current progress of non-viral gene delivery compared to viral gene delivery and comprehensively described the effect of molecular structure factors of chitosan on gene delivery efficiency, characterization techniques for chitosan–polynucleotide complexes, and gene therapy challenges for rare diseases (the citation number: 22).

Stavroula Nanaki et al. [8] reported the preparation of PLA and poly(lactic-*co*-glycolic acid) (PLGA) microspheres containing paliperidone (an antipsychotic drug)-loaded mesocellular silica foam (MCF) coated with thiolated chitosan (the citation number: 16). They intended to apply the microspheres to the intranasal delivery of the drug due to the advantages of thiolated chitosan for penetration into the nasal mucous membrane. As a similar use of chitosan, Boting Lu et al. [9] reported the preparation of chitosan-modified PLGA nanoparticles and investigation of the efficiency of drug loading, encapsulation and cellular uptake using paclitaxel (an anticancer drug) as a model drug (the citation number: 15). Thennakoon M. Sampath Udeni Gunathilake et al. [10] prepared gas-foamed hydrogels of cellulose nanocrystal-reinforced chitosan (the citation number: 14). They investigated the drug release kinetics of the porous hydrogels using curcumin as a drug.

PLGA is also one of major polymers that have been used for biomaterial applications for several decades. Not so high but steady numbers of papers related to PLGA have been published for these three years (2017–2019). I picked up two papers as follows. Xiaoyu Sun et al. [11] reviewed the development of PLGA application to periodontal tissue regeneration (the citation number: 34). They concluded that PLGA is a promising material for this application and showed future prospects. Ranjith Kumar Kankala et al. [12] reported the application of 3D printing technologies to the fabrication process for porous scaffolds for bone tissue regeneration (the citation number: 24). After the 3D printing process, they coated the scaffold with gelatin and nano-hydroxyapatite by freeze drying to enhance the hydrophilicity and biocompatibility. The biocompatibility assays of this material resulted in the good adhesion and proliferation of osteoblast precursor cells (MC3T3-E1).

3D printing technologies are now spreading wider and wider over many fields. 3D bioprinting is an ideal technology for the fabrication of scaffolds to prepare custom-made bioartificial organs. Xiaohong Wang et al. reviewed literature on 3D bioprinting technologies related to gelatin-based hydrogels [13] (the citation number: 42, according to the Scopus counter; only for this paper, the counter of Web of Science seems not to work adequately for Sep. 2020). The review referred to challenges for the 3D bioprinting of complex organs by utilizing multi-nozzle extrusion-based 3D printers and cell-laden hydrogels.

Here, I introduce three more article papers attracting attention. Katarzyna Zubik et al. [14] developed an interesting wound dressing material using poly(*N*-isopropylacrylamide) and cellulose nanocrystals without any additional crosslinkers (the citation number: 34). They showed the thermo-responsiveness and drug loading-releasing ability of the hybrid hydrogels as well as the possibility to apply them as injectable hydrogels for wound dressing. As an emerging application of lignin, Mahboubeh Pishnamazi et al. [15] applied carboxylated lignin to the pH-dependent release of paracetamol (an anti-inflammatory drug) (the citation number: 34). An increasing release rate was shown at pH 7.2, compared to the rate at pH 1.2. Huan Guo et al. [16] investigated the chemical structure and the optimum extraction method for polysaccharides from snow chrysanthemum (*Coreopsis tinctoria*), which is known not only as a popular tea material but also as a traditional medicine (the citation number: 23). They analyzed the monomer constituents of polysaccharides extracted by three methods: simple immersion in hot water, ultra-sonication and microwave irradiation. They found high antioxidant activities for polysaccharides extracted by microwave-assisted extraction.

## 3. Category (2): Environmentally Benign Polymers

In this category, the keywords “PLA” and “composite” can be often seen, but no predominant keyword was found. The most cited paper in this category is a review paper titled “The Recent Developments in Biobased Polymers toward General and Engineering Applications: Polymers that are Upgraded from Biodegradable Polymers, Analogous to Petroleum-Derived Polymers, and Newly Developed” co-authored by Hajime Nakajima, Peter Dijkstra and Katja Loos [17] (the citation number: 79). Impressively, they showed the development of biobased polymers in Table 1 of their literature by dividing the applications of biobased polymers into four categories: super-engineering applications, engineering/semi-engineering applications, general applications and biodegradable/biocompatible applications. Furthermore, in the last section, they introduced newly developed biobased polymers such as poly(ethylene 2,5-frandicarboxylate) (PEF), polyterpenes and biobased liquid crystal polymers (LCP).

The next most cited paper is a review paper titled “Poly(lactic acid) Composites Containing Carbon-Based Nanomaterials: A Review” co-authored by Carolina Gonçalves, Inês C. Gonçalves, Fernão D. Magalhães and Artur M. Pinto [18] (the citation number: 46). Nanocarbon materials (carbon-based nanomaterials) such as carbon nanotubes and graphene-based materials have been tested as filler materials for industrial polymers. The authors carefully collected and summarized references addressing composite materials of PLA and carbon-based nanomaterials. A review paper titled “Bio-Based Adhesives and Evaluation for Wood Composites Application” co-authored by Fatemeh Ferdosian, Zihe Pan, Guchuhan Gao and Boxin Zhao [19] is also attracting citations (the citation number: 45). In this review, the authors reviewed recent studies on biobased wood adhesives from the point of view of materials such as lignin, starch and plant proteins as well as from the point of view of evaluation methods for the wood adhesive performance.

When I searched for papers with the keyword “PLA” using the online search system of *Polymers*, a lot of papers were returned: 26 papers published in 2017 (Volume 9), 60 papers in 2018 (Volume 10) and 98 papers published in 2019 (Volume 11). As a research article, an article titled “Morphology, Crystallization and Thermal Behaviors of PLA-Based Composites: Wonderful Effects of Hybrid GO/PEG via Dynamic Impregnating” co-authored by Shikui Jia, Demei Yu, Yan Zhu, Zhong Wang, Ligui Chen and Lei Fu [20] obtained a lot of citations (the citation number: 37). The “dynamic impregnating” technology was previously developed by the authors. It is a combined technology of vacuuming impregnation under supersonic vibration. They prepared the graphene oxide (GO)/polyethylene glycol (PEG) hybrid at first, and subsequently prepared GO/PEG/PLA composites. They observed the improvement of the storage modulus and heat distortion temperature of PLA. Teboho C. Mokhena et al. [21] overviewed preparation and processing methods, and the mechanical, dynamic mechanical and thermal properties of PLA reinforced with cellulose nanomaterials (cellulose nanocrystals and cellulose nanofibers) (the citation number: 25). This type of material is known as “green composites”, which are of increasing concern due to their sustainability. Jingyao Sun et al. [22] introduced each piece of technology for the fabrication of nanofiller-reinforced nanocomposites and topics on nanocomposite applications (the citation number: 24), though this review paper is not comprehensive. Edgar Castro-Aguirre et al. [23] investigated the influence of nanoclays on the biodegradation behavior of PLA nanocomposites under composting conditions (the citation number: 23). They also checked the influence of a quaternary ammonium salt that is used as an organic surfactant modifier to improve interface compatibility between polymers and clays. Luigi Botta et al. [24] reported an experimental study on the rheological, thermal and mechanical properties of reprocessed PLA/graphene nanoplatelet (GNP) nanocomposites (the citation number: 21). They evaluated the effect of reprocessing on the properties of the nanocomposites to simulate mechanical recycling. Miguel Ángel Caminero et al. [25] investigated the mechanical properties, dimensional accuracy and flatness of the surface of PLA/GNP manufactured by 3D printing with fused filament fabrication (the citation number: 21).

By widening the searched area from PLA to biodegradable or biobased polyesters, some other articles were found. A short review authored by Roberto Scaffaro et al. [26] addresses the biodegradability and recyclability of aliphatic polyesters (the citation number: 21). Another short review was authored by Shen Su et al. [27]. It briefly reviews literature on the toughness, crystallization behaviors, biodegradability and recyclability of PLA/poly(butylene succinate) (PBS) blends (the citation number: 15). Biobased polyesters synthesized using 2,5-furandicarboxylic acid (FDCA) with diols were recently developed as sustainable substitutes for industrial polyesters such as poly(ethylene terephthalate). FDCA is chemically derived from renewable resources such as hexose and therefore cellulose, hemicellulose and starch. Lucia Maini et al. [28] reported the determination of the crystal structure of PEF by fitting the X-ray diffraction powder pattern (the citation number: 26). Atomic coordinates are also reported for the polymorphs of α-, α′- and β-PEF. Jinggang Wang et al. [29] reported the properties of biobased polyesters synthesized from FDCA, conventional diols (ethylene glycol, 1,3-propanediol or 1,4-butanediol) and 2,2,4,4-tetramethyl-1,3-cyclobutanediol (CBDO) (the citation number: 24). CBDO is a co-monomer with a cyclic structure used to improve the physical properties of corresponding biobased polyesters. Indeed, the glass transition temperature, tensile modulus and strength increased upon the incorporation of CBDO.

Besides papers related to polyesters, we can see some papers related to carbohydrate polymers and plant oils. Especially, papers related to cellulose and starch are high in number. As papers addressing cellulose nanofibers or cellulose nanocrystals relate to a large area of this section, here, I introduce two papers that reported the isolation of cellulose microfibrils and cellulose nanocrystals from renewable materials. Monika Szymańska-Chargot et al. [30] prepared and characterized cellulose microfibrils from various fruit and vegetable pomaces (the citation number: 28), while Buong Woei Chieng et al. [31] prepared and characterized cellulose nanocrystals from oil palm mesocarp fibers (the citation number: 28).

Among articles related to starch, a research article titled “How Glycerol and Water Contents Affect the Structural and Functional Properties of Starch-Based Edible Films” co-authored by Ewelina Basiak, Andrzej Lenart and Frédéric Debeaufort [32] is attracting relatively a lot of citations (the citation number: 31). Thermoplastic starch is also one of the major biobased and biodegradable polymers. From the point of view of edible film, they utilized glycerol as a plasticizer and investigated the thermal properties, surface properties, hydration properties, mechanical properties, and water vapor and oxygen permeabilities. If starch films are applied for food packaging, not as edible films, antimicrobial activity is needed to keep food fresh and safe. Zhijun Wu et al. [33] developed antimicrobial films of a starch/poly(vinyl alcohol)/citric acid ternary blend (the citation number: 29).

Papers related to chitosan are not frequently seen in this category, because the amount of industrial supply is not so large. However, I found one review attracting citations titled “Recent Advances of Chitosan Applications in Plants” co-authored by Massimo Malerba and Raffaella Cerana [34] (the citation number: 34). This paper reviews the effect of chitosan on plant growth, pathogen resistance and the commercial life of fruits after detachment.

Polymers derived from vegetable oils or essential oils are categorized as another type of biobased polymer. Adrián Tenorio-Alfonso et al. [35] reported the preparation of novel biobased polyurethane adhesives synthesized from partially acetylated cellulose, castor oil and 1,6-hexamethylene diisocyanate with various contents of castor oil (the citation number: 25). The adhesion properties were evaluated with mechanical tests for bonding wood. Puyou Jia et al. [36] reported the preparation of cardanol group-grafted poly(vinyl chloride) (PVC) as internally plasticized PVC (the citation number: 26). Cardanol is a main component of cashew nut shell liquid. The modification was carried out by the Click reaction between azide-functionalized PVC and cardanol propargyl ether.

## 4. Category (3): Biomacromolecules for Other Applications or General Science and Technology of Biomacromolecules

For this category, I gathered papers addressing biomacromolecules, biobased polymers and biodegradable polymers whose applications were considered to be widely spread in the biomaterial, biomedicine and environmentally benign material fields, and papers addressing other applications. Especially, PLA and cellulose are used widely in all fields of biomaterials, biomedicine and environmentally benign materials. At first, I introduce two review papers on cellulose here. A review paper titled “Cellulose Aerogels: Synthesis, Applications, and Prospects” was co-authored by Lin-Yu Long, Yun-Xuan Weng and Yu-Zhong Wang [37] (the citation number: 46). Aerogel is a porous material with a low density and high porosity, and it is expected to be applied widely in a lot of fields, such as biomedicine, thermal insulation, optoelectronics and oil/water separation. They summarized the preparation methods, properties and applications of cellulose aerogels, categorizing them into three types: natural cellulose aerogels (nanocellulose and bacterial cellulose), regenerated cellulose aerogels and cellulose derivative aerogels. A comprehensive review paper titled “Recent Advances in Nanocellulose Composites with Polymers: A Guide for Choosing Partners and How to Incorporate Them” was co-authored by Arindam Chakrabarty and Yoshikuni Teramoto [38] (the citation number: 44). They described recent approaches to improving the interaction between polymers and nanocellulose in detail. At the end of the review, they discussed the outlook for these nanocomposite materials from an industrial point of view.

Two papers on PLA are introduced here as papers related to many fields. First, a review titled “Chemical Modification and Foam Processing of Polylactide (PLA)” co-authored by Tobias Standau, Chunjing Zhao, Svenja Murillo Castellón, Christian Bonten and Volker Altstädt [39] summarizes technologies for the foam processing of PLA with comprehensible depictions (the citation number: 24). PLA foam is expected to expand in application to wider fields. Michał Puchalski, Sylwia Kwolek, Grzegorz Szparaga, Michał Chrzanowski and Izabella Krucińska investigated the crystalline forms and mechanical properties of wet-spun PLA in a scientific paper titled “Investigation of the Influence of PLA Molecular Structure on the Crystalline Forms (α’ and α) and Mechanical Properties of Wet Spinning Fibres” [40] (the citation number: 23). They found that the molecular weights and D-lactide contents influenced spinning parameters such as the draw ratio and therefore the crystalline forms and mechanical properties.

Carbohydrate polymers (polysaccharides) other than cellulose and starch are also investigated and reviewed often from pure scientific interest without specific applications. Hyaluronic acid (HA) is a copolymer of d-glucuronic acid and *N*-acetyl-d-glucosamine linked by β-1,3 glycosidic bonds. HA exists in the connective, epithelial and nervous tissues of vertebrates. A review paper titled “Hyaluronic Acid in the Third Millennium” was co-authored by Arianna Fallacara, Erika Baldini, Stefano Manfredini and Silvia Vertuani [41] (the citation number: 44). The review summarizes widely accumulated knowledge on HA like a textbook, and furthermore, the explanation of HA’s applications is well-written. Pectins are also carbohydrate polymers mainly composed of D-galacturonic acid found in the primary cell walls of higher plants. Diana Gawkowska, Justyna Cybulska and Artur Zdunek [42] reviewed pectin gelation behaviors in a review titled “Structure-Related Gelling of Pectins and Linking with Other Natural Compounds: A Review” (the citation number: 28). It summarizes the gelation mechanisms of pectin from the point of view of the chemical structures of each pectin family. Crosslinking agents and the interaction of pectins with other natural compounds are also referred to in the review paper. In contrast to these two reviews, Cong Wang et al. [43] summarized a more specific application of carbohydrate polymers for the preparation of metallic nanoparticles (MNP) (the citation number: 23). The authors introduced the role of carbohydrate polymers in the bottom-up synthesis of MNP as a host material, and focused on the applications of polysaccharide-based MNP.

Except for PLA and carbohydrate polymers, the following paper related to a plant protein gathers a moderately high number of citations: “The Relationships between the Working Fluids, Process Characteristics and Products from the Modified Coaxial Electrospinning of Zein” co-authored by Menglong Wang, Tao Hai, Zhangbin Feng, Deng-Guang Yu, Yaoyao Yang and S.W. Annie Bligh [44] (the citation number: 38). Zein is a protein extracted from maize and attracting attention as a protein material available for industrial application. Though the authors selected zein as a model system, this article provides important information on the electrospinning of zein as well as on coaxial electrospinning, which is a recently developed technology for the preparation of core-sheath electrospun nanofibers by ejecting two fluids together from a single spinneret in a coaxial manner.

As the last section, I introduce two additional papers addressing applications other than biomaterial, biomedicine and environment-related fields. Shujahadeen B. Aziz, Mariwan A. Rasheed and Hameed M. Ahmed reported an advanced use of a cellulose derivative in an article titled “Synthesis of Polymer Nanocomposites Based on [Methyl Cellulose]_(1 − *x*)_:(CuS)_x_ (0.02 M ≤ *x* ≤ 0.08 M) with Desired Optical Band Gaps” [45] (the citation number: 22). In this report, methyl cellulose (MC) is used as a matrix of CuS semiconductor nanoparticles, and the band gap decreased and the refractive index increased with an increase in the CuS fraction. The increase in the refractive index of the nanocomposite was linear, indicating a homogeneous dispersion of CuS nanoparticles. Yao Huang et al. [46] comprehensively reviewed recent developments of conductive polymer composites, mainly from natural polymers and conductive fillers such as nanocarbon materials and metallic or metal oxide particles (the citation number: 18). The authors summarized the preparative methods, properties and potential applications of these composite materials.

## 5. Outlook

Analyzing the numerous papers published in the “Biomacromolecules, Biobased and Biodegradable Polymers” section in 2017–2019, we can see the research trends in this field. For biomedicinal applications, chitosan is predominantly selected as a base polymer or a modifying polymer. On the other hand, we can see only a few well-cited papers dealing with collagen and gelatin. As for application fields, drug delivery systems and controlled release have been studied for several decades and are still often picked up. According to the impression of recent tendencies, 3D printing technologies have been emerging in this decade, perhaps due to the popularization of 3D printing technologies. The technology now covers not only industrial manufacture but also biomaterial fabrication (3D bioprinting).

As for industrial applications, thermoplastic aliphatic polyesters have been a trend in environmentally benign materials for several decades, and more recently, those reinforced with nanocellulose and nanocarbon materials are often reported and well-referenced. This is also due to the development of technologies for the preparation and analysis of nanosized materials.

In this editorial, I picked up papers considering the numbers of citations. Though this process can show the current trends, I believe that it does not always show the future trends. Therefore, I am expecting that some papers that were not picked up here may change the stream of polymer science and become a seed for future trends.
